# Thiocholine-Mediated One-Pot Peptide Ligation and Desulfurization

**DOI:** 10.3390/molecules28093655

**Published:** 2023-04-22

**Authors:** Sae Suzuki, Yuya Nakajima, Naoki Kamo, Akihisa Osakabe, Akimitsu Okamoto, Gosuke Hayashi, Hiroshi Murakami

**Affiliations:** 1 Department of Biomolecular Engineering, Graduate School of Engineering, Nagoya University, Furo-cho, Chikusa-ku, Nagoya 464-8603, Japan; 2 Department of Chemistry and Biotechnology, Graduate School of Engineering, The University of Tokyo, 7-3-1 Hongo, Bunkyo-ku, Tokyo 113-8656, Japan; 3 Department of Biological Sciences, Graduate School of Science, The University of Tokyo, 7-3-1 Hongo, Bunkyo-ku, Tokyo 113-0033, Japan; 4 Institute of Nano-Life-Systems, Institutes of Innovation for Future Society, Nagoya University, Furo-cho, Chikusa-ku, Nagoya 464-8603, Japan

**Keywords:** chemical protein synthesis, native chemical ligation, desulfurization, one-pot synthesis

## Abstract

Thiol catalysts are essential in native chemical ligation (NCL) to increase the reaction efficiency. In this paper, we report the use of thiocholine in chemical protein synthesis, including NCL-based peptide ligation and metal-free desulfurization. Evaluation of thiocholine peptide thioester in terms of NCL and hydrolysis kinetics revealed its practical utility, which was comparable to that of other alkyl thioesters. Importantly, thiocholine showed better reactivity as a thiol additive in desulfurization, which is often used in chemical protein synthesis to convert Cys residues to more abundant Ala residues. Finally, we achieved chemical synthesis of two differently methylated histone H3 proteins via one-pot NCL and desulfurization with thiocholine.

## 1. Introduction

Native chemical ligation (NCL) between N-terminal cysteinyl peptide and C-terminal peptide thioester [1] is a key reaction in chemical protein synthesis [2,3,4], and it commonly exploits thiol catalysts to enhance the reaction kinetics [5]. If the thiol proton of an externally added catalyst has a lower pKa than that of the thiol compound incorporated in the original peptide thioester, more activated thioester species are generated through thiol–thioester exchange [6]. Among several available thiol additives, 4-mercaptophenyl acetic acid (MPAA) has been most widely used in NCL-based peptide ligation [7] due to its sufficiently low pKa (6.6) and highly hydrophilic nature at neutral pH. However, when NCL is followed by free-radical desulfurization [8], where Cys residues are converted to more abundant Ala residues [9], MPAA causes an inhibitory effect by trapping the radical species. Compared to MPAA and other aryl thiols, alkyl thiols such as 2-mercaptoethyl sulfonate (MESNa) [7,10] have relatively high pKa values (9.2), leading to stable thioester moieties that become suitable for isolating peptide thioesters. Notably, alkyl thiols are compatible with free-radical desulfurization, thus allowing one-pot NCL and desulfurization [11,12,13]. As desulfurization-compatible alkyl thiols with relatively low pKa values, methyl thioglycolate (MTG, pKa = 7.9) [14], trifluoroethanethiol (TFET, pKa = 7.3) [6,15], and 2-sulfanylmethyl-4-dimethylaminopyridine (SMDMAP, pKa = 6.15) [16] have been developed so far.

**Figure 1:**
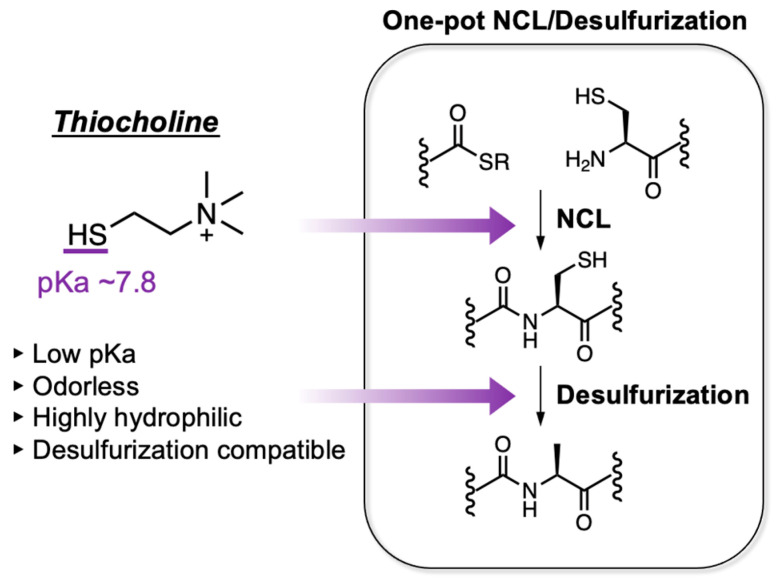
Characteristics of thiocholine and its use in one-pot NCL/desulfurization.

In this study, we report NCL-based peptide ligation and desulfurization using thiocholine as a thiol additive. Thiocholine is an odorless and highly hydrophilic compound bearing an alkyl thiol group with a pKa of ~7.8 at room temperature [17]; therefore, it was expected to be useful as a peptide thioester or thiol additive in NCL and desulfurization (Figure 1). We evaluated the thiocholine peptide thioester in terms of hydrolysis resistance and NCL reactivity by comparing it with other alkyl thioesters. Then, the use of thiocholine as an additive in desulfurization was also examined. Finally, we semisynthesized histone H3 proteins tethering lysine dimethylation or trimethylation by thiocholine-mediated one-pot NCL/desulfurization reaction.

## 2. Results and Discussion

### 2.1. Synthesis of Thiocholine Peptide Thioester

Commercially available acetylthiocholine iodide was acid-hydrolyzed to quantitatively obtain thiocholine chloride (Scheme S1) according to the previous literature [18]. Then, a model peptide **1** bearing C-terminal thiocholine thioester was synthesized to investigate its hydrolysis resistance and NCL reactivity. First, a heptapeptide bearing C-terminal hydrazide was synthesized via standard 9-fluorenylmethoxycarbonyl (Fmoc) solid-phase peptide synthesis (SPPS) with 59% isolated yield and >98% purity (Figure S1A). Then, the hydrazide moiety was converted to thioester by NaNO_2_ activation [19,20] followed by thiocholine treatment to afford thiocholine peptide thioester **1** (41% isolated yield and >95% purity) (Figure S1B). In the same procedure, four other different peptide thioesters composed of TFET, MTG, MESNa, and thioglycolic acid (TGA) were also synthesized to determine the relative properties of thiocholine thioester (Figure S1C–F). Notably, the peptide thioesters used here had a C-terminal Gly residue, which is known to have higher NCL reactivity and lower hydrolytic resistance than other C-terminal amino acid residues [15,21].

### 2.2. Hydrolytic Stability and NCL Reactivity of Thiocholine Thioester

**Figure 2:**
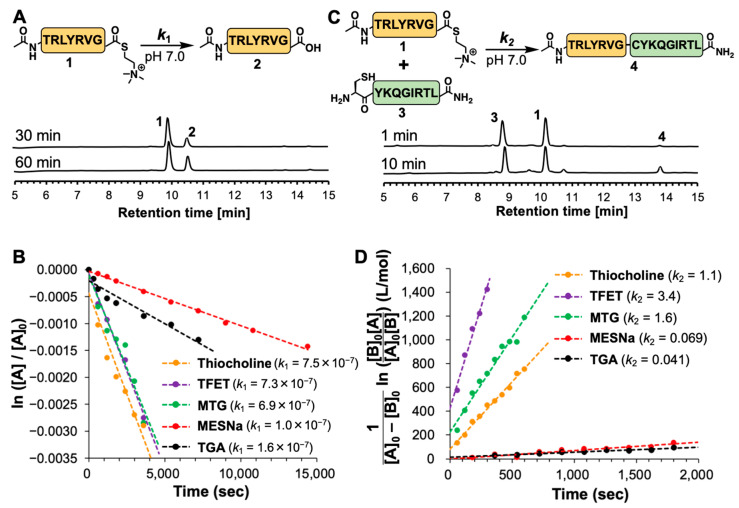
Hydrolytic stability and NCL reactivity of thiocholine and other peptide thioesters. (**A**) Reaction scheme and time-course HPLC analysis of hydrolysis reaction of thiocholine peptide thioester. (**B**) Determination of pseudo first-order reaction rate constants of hydrolysis reaction for peptide thioesters (thiocholine, TFET, MTG, MESNa, and TGA). Kinetic constants of hydrolysis reactions (*k*_1_) were determined using the following equation: ln ([A]/[A]_0_) = −*k*_1_t. (**C**) Reaction scheme and time-course HPLC analysis of NCL reaction of thiocholine peptide thioester. (**D**) Determination of second-order reaction rate constants of NCL reaction for peptide thioesters (thiocholine, TFET, MTG, MESNa, and TGA). Kinetic constants of NCL reactions (*k*_2_) were determined using the following equation: [1/([A]_0_ − [B]_0_)] ln ([B]_0_[A]/[A]_0_[B]) = *k*_2_t.

To evaluate the hydrolytic stability of thiocholine thioester, peptide **1** (1 mM) was dissolved in NCL buffer containing 200 mM NaH_2_PO_4_ (pH 7.0), 6 M Gn·HCl, and 40 mM TCEP, incubated at 25 °C, and analyzed by reversed-phase HPLC. The degree of hydrolysis gradually increased and reached 35% at 1 h (Figure 2A). The pseudo first-order rate constant *k*_1_ of the hydrolysis was estimated as 7.5 × 10^−7^ s^−1^ by determining the concentration of hydrolyzed peptide **2** at each time point using a calibration curve (Figure 2B and Figure S2A,D and S3). In the same analysis of the four other peptide thioesters, TFET and MTG thioesters showed similar *k*_1_ values of 7.3 × 10^−7^ and 6.9 × 10^−7^ s^−1^, respectively, whereas MESNa and TGA thioesters showed relatively slower hydrolytic rates (*k*_1_ = 1.0 × 10^−7^ and 1.6 × 10^−7^ s^−1^, respectively), suggesting that the hydrolysis rates were consistent with their pKa values.

We then investigated NCL reactivity of the peptide thioesters with N-terminal Cys peptide **3** prepared by standard Fmoc SPPS (Figure S2B). Thiocholine thioester peptides **1** and **3** were mixed in NCL buffer at 25 °C, and analyzed by reversed-phase HPLC. Notably, the peptide concentration was set at 0.1 mM, which was lower than a conventional condition around 1 mM, in order to make the initial reaction rate easily detectable. Time-course HPLC analysis showed that the ligated peptide **4** gradually appeared and reached 7.6% after 10 min (Figure 2C). The apparent second-order rate constant *k*_2_ was estimated as 1.1 M^−1^s^−1^ by determining the concentration of ligated peptide **4** at each time point using a calibration curve (Figure 2D and Figures S2C,E and S4). TFET and MTG thioesters showed relatively higher *k*_2_ values of 3.4 and 1.6 M^−1^s^−1^, respectively, whereas MESNa and TGA thioesters showed significantly slower NCL rates (*k*_2_ = 6.9 × 10^−2^ and 4.1 × 10^−2^ M^−1^s^−1^, respectively).

These two experiments revealed that the thiocholine thioester could be classified as a reactive alkyl thioester, such as TFET and MTG thioesters, and its reactivity corresponded to the p*K*_a_ value of thiocholine. Therefore, we reasoned that thiocholine would be useful as a thiol catalyst in NCL-based peptide ligation.

### 2.3. Desulfurization with Thiocholine

**Figure 3:**
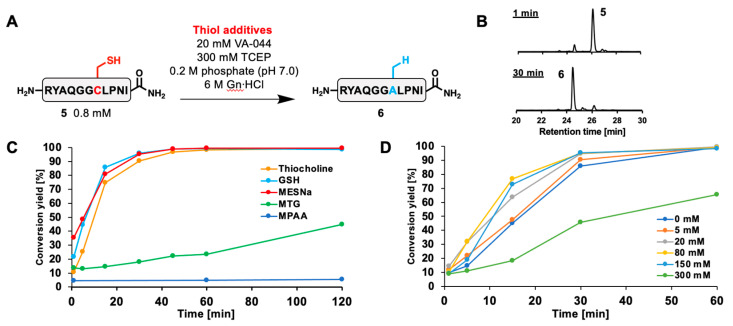
Free radical desulfurization of model peptide **5** with thiol additives. (**A**) Reaction scheme of desulfurization of model peptide **5**. (**B**) HPLC profiles of thiocholine-mediated desulfurization. (**C**) Time-course analysis of desulfurization with 80 mM thiol additives (thiocholine, GSH, MESNa, MTG, or MPAA). (**D**) Time-course analysis of desulfurization with different thiocholine concentrations. The conversion yields were determined by HPLC peak areas (220 nm) at each time point.

Thiol additives are usually used in free-radical desulfurization reactions for efficient propagation of radical species [8,22,23,24]. We investigated the reactivity of thiocholine as a thiol additive in the desulfurization-mediated Cys-to-Ala conversion. First, an internal Cys-containing model peptide **5** was synthesized via standard Fmoc SPPS (Figure S5). Then, peptide **5** was desulfurized in the presence of 80 mM thiocholine, 20 mM VA-044, and 300 mM TCEP (Figure 3A). After reaction for 1 h, starting peptide **5** was almost quantitatively converted to desulfurized peptide **6** (Figure 3B and Figure S6). When the desulfurization rate was compared with that of other thiol additives, thiocholine, MESNa, and glutathione (GSH) exhibited similar fast kinetics, whereas MTG and MPAA showed significantly slower rates (Figure 3C and Figure S7). Notably, the reaction mixture containing MTG reached completion within several hours, while desulfurization with MTG is usually performed with additional alkyl thiol additives presumably to accelerate the reaction rate [14,25,26,27]. We assume that the slow reaction rate of MTG could be caused by the stable alkyl radical generated after the TCEP-mediated sulfur atom abstraction. This alkyl radical should be stabilized by resonance effects with its carbonyl group and the energy barrier for the subsequent hydrogen atom abstraction might be high [28]. On the other hand, MPAA did not reach completion after a few days as previously reported [11]. Next, the concentration dependence of the thiol additive was examined with/without 5, 20, 80, 150, and 300 mM thiocholine (Figure 3D and Figure S8). Surprisingly, the desulfurization reaction reached completion without any thiol additive after 1 h, suggesting that external thiol-mediated radical propagation would not be an essential step in desulfurization as recently reported [29,30,31]. The presence of 20–150 mM thiocholine moderately accelerated the reaction rates and achieved full conversion. On the other hand, 300 mM thiocholine significantly reduced the reaction rate. This was probably due to the deficiency of TCEP, which is necessary to abstract sulfur atoms from thiol moieties of Cys or thiol additives in the course of the desulfurization mechanism [30]. 

### 2.4. One-Pot NCL/Desulfurization for Histone H3K4me3 Semisynthesis

**Figure 4:**
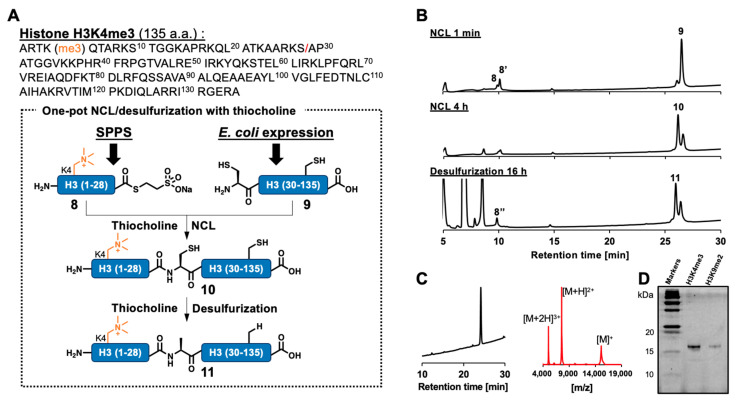
Semisynthesis of Arabidopsis H3K4me3 via thiocholine-mediated one-pot NCL/desulfurization. (**A**) Amino acid sequence and synthetic scheme of H3K4me3. A red slash represents the ligation junction. (**B**) Reaction tracking of one-pot NCL/desulfurization by analytical HPLC (gradient: 5–75% for 30 min) at 220 nm. Peptides **8′** and **8″** indicate thiocholine thioester and hydrolyzed **8**, respectively. HPLC profiles of NCL at 1 min, NCL at 4 h, and desulfurization at 16 h are shown. (**C**) HPLC profile (left, gradient 20–70% for 30 min) and MALDI-TOF mass spectrum (right) of purified peptide **11**. Calculated mass of peptide **11** [M]^+^, 15,147.5; mass found [M]^+^, 15,146.8. (**D**) SDS-PAGE gel of purified H3K4me3 (peptide **11**) and H3K9me2 (peptide **15**, in Figure S13).

To demonstrate the utility of thiocholine-mediated NCL and desulfurization in chemical protein synthesis, we conducted one-pot semisynthesis of Arabidopsis trimethylated histone H3 (H3K4me3), which is related to transcriptional activation [32,33,34,35,36]. The full-length H3 sequence (135 aa) was divided into two peptide segments at the Ser28–Ala29 junction (Figure 4A). The N-terminal segment **8** was synthesized as MESNa thioester, which was more stable than thiocholine thioester and suitable for isolating peptide thioester, via NaNO_2_ activation of C-terminal hydrazide peptide **7** (Figure S9). The C-terminal 107 aa segment was prepared as an N-terminal Cys peptide through *E. coli* expression of N-terminally His-tagged protein and following tag removal by enterokinase cleavage. The recovered crude peptide **9** was purified by ion exchange chromatography, treated with methoxyamine to open the thiazolidine ring [37], and finally purified by HPLC (Figure S10).

Peptide ligation was conducted with peptide thioester **8** and N-terminal Cys peptide **9** in NCL buffer containing 80 mM thiocholine and monitored by HPLC (Figure 4B). Within one minute, MESNa thioester **8** was converted to thiocholine thioester **8′** and ligation product **10** was observed as a main peak at 4 h (Figure S11). Then, radical initiator VA-044 and sulfur scavenger TCEP were added to start desulfurization in a one-pot manner. After 16 h, the observed main peak in the HPLC chart was fractionated and purified. As a result, desulfurized product **11**, full-length H3K4me3, was successfully obtained with high purity and identified by MALDI-TOF mass spectrometry (Figure 4C). In the same thiocholine-mediated one-pot NCL/desulfurization strategy, we also completed the synthesis of Arabidopsis H3K9me2 (Figures S12 and S13), which is known to be correlated with gene silencing [34,38,39,40,41,42,43]. SDS-PAGE of full-length H3K4me3 and H3K9me2 confirmed that these products were obtained with high purity (Figure 4D).

## 3. Materials and Methods

### 3.1. General

All Fmoc or Boc protected amino acids were purchased from Watanabe Chemical (Osaka, Japan), Novabiochem (San Diego, CA, USA), or CEM, Inc. (Tokyo, Japan). Other chemicals were purchased from Sigma-Aldrich (St. Louis, MO, USA), Wako Chemicals (Tokyo, Japan), Tokyo Chemical Industry (Tokyo, Japan), or other commercial suppliers. All solvents and reagents were used without further purification. NMR spectra were recorded with an UltraShield 300 MHz (Bruker, Billerica, MA, USA). MALDI-TOF MS measurements were performed using an AutoFlex Max (Bruker). Preparative HPLC purification of the peptides was carried out using an MD-4010 Photodiode Array Detector at 190 to 900 nm (JASCO, Tokyo, Japan), UV-4075 UV/Vis Detector at 190 to 600 nm (JASCO), PU-4180 HPLC Pump (JASCO), and the 5C_18_ AR-II column (20 ID × 250; Nacalai, Kyoto, Japan), Protein-R column (20 ID × 250; Nacalai), or Jupiter 5C_4_ 300Å column (21.2 ID × 250; Phenomenex, Torrance, CA, USA) with a binary mixture of A (water containing 0.1% trifluoroacetic acid [TFA]) and B (acetonitrile containing 0.1% TFA) as the mobile phase (flow rate = 7.5 mL/min) in a linear gradient. For the analytical HPLC measurements of the peptides, the 5C_18_ AR-II column (4.6 ID × 250; Nacalai), Protein-R column (4.6 ID × 250; Nacalai), or Jupiter 5C_4_ 300 Å column (4.6 ID × 250; Phenomenex) was used with a binary mixture of A and B as the mobile phase (flow rate = 1.0 mL/min) in a linear gradient, as described in each figure or protocol. All HPLC charts indicated in this paper were monitored at 220 nm. All heating protocols were conducted in the COOL-INCUBATOR HCRCS2V75W-A0602 (IKUTA Sangyo, Kawasaki City, Japan).

### 3.2. Synthesis of Thiocholine Chloride

Thiocholine chloride was synthesized following previously described procedures [18,44]. Briefly, acetylthiocholine iodide, 2-(acetothioethyl)-trimethylammonium iodide (600 mg, 2.07 mmol), was added to a 50 mL round bottom flask and dissolved in degassed 4 N HCl (5 mL) under argon atmosphere. The stirred solution was heated to 85 °C. After 2 h, the reaction mixture was concentrated and dried under reduced pressure to provide a pale yellow solid. The solid was dissolved in MeOH (~5 mL) and recrystallized at 4 °C to provide the product thiol (thiocholine chloride) as a very hygroscopic solid (321 mg, quant.). This material was used without further purification. 1H NMR (D_2_O, 300 MHz): δ = 2.88 (2H, m, CH_2_), 3.06 (9H, s, NMe_3_), 3.45 (2H, m, CH_2_).

### 3.3. Synthesis of Model Peptides

#### 3.3.1. General Method of Peptide Synthesis

Automated solid-phase peptide synthesis was performed using ResPep CF (Intavis, Tübingen, Germany), Liberty Blue (CEM), or Initiator+ Alstra (Biotage, Uppsala, Sweden) via the standard Fmoc-SPPS protocol. ResPep CF: Fmoc-protected amino acids (5.2 eq.) were coupled with O-(1H-benzotriazol-1-yl)-N,N,N′,N′-tetramethyluronium hexafluorophosphate (HBTU; 5.0 eq.) and N,N-diisopropylethylamine (DIEA; 10 eq.). Liberty Blue: Fmoc-protected amino acids (4.0 eq.) were coupled with N,N′-diisopropylcarbodiimide (DIC; 8.0 eq.) and ethyl (hydroxyimino)cyanoacetate (4.0 eq.; OxymaPure). Initiator+ Alstra: Fmoc-protected amino acids (4.0 eq.) were coupled with HBTU (3.92 eq.) and DIEA (8.0 eq.). The isolated yields of each peptide were estimated using the molecular weights of TFA salt at the N-terminal amine and sidechains of Arg, Lys, Lys(me)_2_, Lys(me)_3_, and His.

#### 3.3.2. Synthesis of Model Peptide Thioesters

C-terminal peptide hydrazide, Ac-TRLYRVG-NHNH_2_ was first synthesized with Cl-Trt(2-Cl)-Resin (Watanabe Chemical, 1.34 mmol/g, 200 μmol scale). After swelling the resin in DMF for 20 min, Fmoc-hydrazine (50.8 mg, 200 μmol) and DIEA (96.8 μL, 0.558 mmol) in DMF (12 mL) were added to the resin, and the mixture was agitated at room temperature for 16 h. After Fmoc quantification, Fmoc-SPPS was conducted using Initiator+ Alstra. After elongation of the peptide chain, the N-terminal Fmoc group was removed manually by adding 20% piperidine in DMF twice for 5 min each, and the N-terminal free amino group was acetylated in 25% Ac_2_O/CH_2_Cl_2_ for 5 min. After washing the resin with DMF and CH_2_Cl_2_, a cleavage cocktail (95.0% TFA, 2.5% triisopropylsilane [TIPS], and 2.5% H_2_O) was added to the reaction column and shaken at room temperature for 2 h. Then, the solution was filtered to remove the resin, and 10 times the volume of cold diethyl ether was added to the filtered solution to precipitate the peptide. This suspension was vortexed and centrifuged at 10,000× *g* at 3 °C for 5 min. Ether was removed by decantation. The precipitate was washed with diethyl ether twice. The crude peptide was dissolved in a water/acetonitrile mixture containing 0.1% TFA, purified by preparative HPLC, and identified by MALDI-TOF mass spectrometry (MS). After lyophilization, the peptide hydrazide was obtained as a white solid (96.7 mg, 84.3 μmol, 59%). The HPLC profile and MS data after purification are shown in Figure S1.

The obtained peptide hydrazide was dissolved (5.0 mM) in 6 M Gn·HCl and 0.2 M NaH_2_PO_4_ at pH 3.0. The peptide solution was chilled to −15 °C and was added to 5.0 M NaNO_2_ aq. (10 equiv. against peptide). The peptide solution was reacted at −15 °C for 15 min. Then, the reaction mixture was added to thiocholine, TFET, MTG, MESNa, or TGA (50 equiv. against peptide) and the pH was adjusted to 6.5–7.0 with 6 N NaOH aq. The reaction mixture was incubated at 25 °C for 30 min. After completion of the reaction, the peptide solution was diluted with a mixture of water/acetonitrile containing 0.1% TFA, purified by HPLC, and identified by MALDI-TOF MS. After lyophilization, thiocholine peptide thioester 1, TFET thioester, MTG thioester, MESNa thioester, and TGA thioester were obtained as white solids (thiocholine thioester **1**: 8.62 mg, 3.95 μmol, 41%; TFET thioester: 7.57 mg, 7.53 μmol, 61%; MTG thioester: 3.93 mg, 2.15 μmol, 17%; MESNa thioester: 9.09 mg, 7.23 μmol, 44%; and TGA thioester: 1.60 mg, 1.31 μmol, 32%).

#### 3.3.3. Synthesis of Other Model Peptides

Peptide **2**, Ac-TRLYRVG-OH, was synthesized with Cl-Trt(2-Cl)-Resin (Watanabe Chemical, 1.34 mmol/g, 50 μmol scale) and the peptide chain was elongated by ResPep CF (Intavis). N-terminal Fmoc group removal and acetyl capping was conducted manually, as described above. Peptide **3**, H-CYKQGIRTL-NH_2_, was synthesized with Rink Amide-PEG Resin (Watanabe Chemical, 0.21 mmol/g, 50 μmol scale) and the peptide chain was elongated by Liberty Blue (CEM). Peptide **4**, Ac-TRLYRVGCYKQGIRTL-NH_2_, was synthesized with Rink Amide-PEG Resin (Watanabe Chemical, 0.21 mmol/g, 50 μmol scale) and the peptide chain was elongated by ResPep CF (Intavis). N-terminal Fmoc group removal and acetyl capping was conducted manually, as described above. Peptide **5**, H-RYAQGGCLPNI-NH_2_, was synthesized with Rink Amide Resin (Watanabe Chemical, 0.50 mmol/g, 50 μmol scale) and the peptide chain was elongated by ResPep CF (Intavis). Then, cleavage cocktail 1 (95.0% TFA, 2.5% triisopropylsilane [TIPS], 2.5% H_2_O) or 2 (92.5% TFA, 2.5% TIPS, 5% 1,3-dimethoxybenzene) was added to the reaction column of peptides **2**–**4** or **5** and shaken at room temperature for 2–3 h. Following ether precipitation, HPLC purification and identification by MALDI-TOF MS was performed, as described above. After lyophilization, each peptide was obtained as a white solid (3.38 mg, 2.98 μmol, 5% for peptide **2**; 17.5 mg, 12.3 μmol, 24% for peptide **3**; 3.04 mg, 1.26 μmol, 2% for peptide **4**; and 35.3 mg, 24.9 μmol, 50% for peptide **5**).

### 3.4. Evaluation of Peptide Thioesters

#### 3.4.1. Determination of Calibration Curves for Peptides **2** and **4**

Serially diluted aqueous solution of peptide **2** or **4** (250, 125, 62.5, 31.25, and 15.625 μM) were prepared in NCL buffer (0.2 M NaH_2_PO_4_ [pH 7.0], 6 M Gn·HCl) containing TCEP (40 mM). A 5 μL aliquot of each solution was taken and mixed with 40 μL of acidic NCL buffer (pH 3.0) and 5 μL of 500 mM TCEP aq. (pH 7.0). Then, 40 μL of each sample was injected into an analytical HPLC equipped with a 4.6 ID × 250 mm COSMOSIL 5C_18_-AR-II column (Nacalai tesque, Kyoto, Japan). HPLC peaks were monitored at 220 nm in a linear gradient with water/acetonitrile containing 0.1% TFA (gradient: 15–50% for 30 min). Calibration curves for peptides **2** and **4** were drawn by plotting HPLC peak areas at each concentration, as shown in Figure S2D,E.

#### 3.4.2. Hydrolysis Kinetics of Peptide Thioesters

Each peptide thioester (1.0 mM) and TCEP (40 mM) were dissolved in NCL buffer (0.2 M NaH_2_PO_4_ [pH 7.0], 6 M Gn·HCl) and incubated at 25 °C. A 5 μL aliquot of the reaction solution was taken and quenched with 40 μL of acidic NCL buffer (pH 3.0) and 5 μL of 500 mM TCEP aq. (pH 7.0). Then, 40 μL of each sample was injected into an analytical HPLC equipped with a 4.6 ID × 250 mm COSMOSIL 5C_18_-AR-II column (Nacalai tesque). HPLC peaks were monitored at 220 nm in a linear gradient with water/acetonitrile containing 0.1% TFA (gradient: 15–50% for 30 min). The concentration of hydrolyzed product peptide **2** at each time point was calculated using the standard curve. The pseudo first-order rate constants of hydrolysis (*k*_1_) were determined using a least squares line fitting the following equation: ln ([A]/[A]_0_) = −*k*_1_t.

#### 3.4.3. NCL Kinetics of Peptide Thioesters

To a solution of peptide **3** (final conc., 0.11 mM) and TCEP (final conc., 40 mM) in NCL buffer (0.2 M NaH_2_PO_4_ [pH 7.0], 6 M Gn·HCl; 400 μL) was added a solution of peptide **1** in NCL buffer (final conc., 0.10 mM). The NCL reaction was performed at 25 °C (pH 7.0), and 50 μL of reaction solution was taken and quenched with 400 μL of acidic NCL buffer (pH 3.0) and 50 μL of 500 mM TCEP aq. (pH 7.0). Then, 40 μL of each sample was injected into an analytical HPLC equipped with a 4.6 ID × 250 mm COSMOSIL 5C_18_-AR-II column (Nacalai tesque). HPLC peaks were monitored at 220 nm in a linear gradient with water/acetonitrile containing 0.1% TFA (gradient: 15–50% for 30 min). The concentration of ligated peptide **4** at each time point was calculated using the standard curve. The apparent second-order rate constants of NCL (*k*_2_) were determined using a least squares line fitting the following equation: [1/([A]_0_ − [B]_0_)] ln ([B]_0_[A]/[A]_0_[B]) = *k*_2_t. Considering that some of the peptide thioesters were partially hydrolyzed before starting the experiment, initial concentrations of peptide thioesters (=[A]_0_) were calculated by subtracting the amount of hydrolyzed peptide **2** at 0 min. Since the hydrolysis rate of peptide thioesters was significantly slower than the NCL rate, the concentration of the hydrolyzed peptide **2** was assumed to be constant.

### 3.5. Desulfurization with Model Peptide

#### 3.5.1. Desulfurization with Different Thiol Additives

To peptide **5** (0.28 mg; final conc., 0.8 mM) and TCEP (21.5 mg; final conc., 300 mM) in 200 µL of degassed NCL buffer (6 M Gn·HCl and 0.2 M sodium phosphate, pH 7.0) were added thiol additives (final conc., 80 mM) thiocholine, MESNa, GSH, MTG, or MPAA. To the reaction solution was added 50 µL of VA-044 (100 mM), and the reaction mixture was incubated at 37 °C under an argon atmosphere (pH 7.0). For analysis of each reaction, 20 μL of reaction solution was taken and quenched with 5 µL of ascorbic acid (500 mM) in acidic NCL buffer (pH 3.0), followed by injection into an analytical HPLC equipped with a 4.6 ID × 250 mm COSMOSIL 5C18-AR-II column (Nacalai tesque). HPLC peaks were monitored at 220 nm in a linear gradient with water/acetonitrile containing 0.1% TFA (gradient: 5–30% for 30 min).

#### 3.5.2. Desulfurization with Different Concentrations of Thiocholine

To peptide **5** (0.28 mg; final conc., 0.8 mM) and TCEP (21.5 mg; final conc., 300 mM) in 200 µL of degassed NCL buffer (6 M Gn·HCl and 0.2 M sodium phosphate, pH 7.0) were added 1.10, 4.26, 17.1, 32.0, or 63.9 µL of 1173 mM thiocholine (final conc., 5, 20, 80, 150, or 300 mM). To the reaction solution was added VA-044 (final conc., 20 mM), and the reaction mixture was incubated at 37 °C under an argon atmosphere (pH 7.0). For analysis of each reaction, 20 μL of reaction solution was taken and quenched with 5 µL of ascorbic acid (500 mM) in acidic NCL buffer (pH 3.0), followed by injection into an analytical HPLC equipped with a 4.6 ID × 250 mm COSMOSIL 5C18-AR-II column (Nacalai tesque). HPLC peaks were monitored at 220 nm in a linear gradient with water/acetonitrile containing 0.1% TFA (gradient: 5–30% for 30 min).

### 3.6. Semisynthesis of Histone H3K4me3 and H3K9me2

#### 3.6.1. Synthesis of Histone H3 N-Terminal Peptide Segments

For the preparation of H3 N-terminal peptide segments, C-terminal peptide hydrazides were prepared with Cl-Trt(2-Cl)-Resin (Watanabe Chemical, 1.34 mmol/g, 50 μmol scale). After swelling the resin in DMF for 20 min, to the resin was added Fmoc-hydrazine (14.0 mg, 55.0 μmol) and DIEA (18.9 μL, 0.110 mmol) in DMF (1.0 mL), and the mixture was agitated at room temperature for 16 h. After Fmoc quantification, standard Fmoc-SPPS was conducted using ResPep CF (Intavis) or Liberty Blue (CEM). Coupling of Fmoc-Lys(me)_2_-OH·HCl (Watanabe Chemical) or Fmoc-Lys(me)_3_-OH chloride (Watanabe Chemical) was performed manually using the following protocol: Fmoc-Lys(me)_2_-OH·HCl (3 equiv.) or Fmoc-Lys(me)_3_-OH chloride (3 equiv.) were activated with HBTU (2.9 equiv.) and DIEA (9 or 6 equiv., respectively) in DMF and transferred to the resin (coupling time: 120 min at room temperature). For Fmoc group deprotection, the resin was treated twice with 20% piperidine in DMF for 5 min each.

After elongation of the peptide chain, the N-terminal Fmoc group was removed by adding 20% piperidine in DMF twice for 5 min each. After N-terminal deprotection, a cleavage cocktail (95.0% TFA, 2.5% TIPS, 2.5% H_2_O) was added to the reaction column and shaken at room temperature for 2.5 h. Then, the solution was filtered to remove the resin, and 10 times the volume of cold diethyl ether was added to the filtered solution to precipitate the peptide. This suspension was vortexed and centrifuged at 10,000× *g* at 3 °C for 5 min. Ether was removed by decantation. The precipitate was washed twice with diethyl ether. Then, the crude peptide was dissolved in a water/acetonitrile mixture containing 0.1% TFA, purified by preparative HPLC, and identified by MALDI-TOF MS. After lyophilization, H3K4me3 peptide hydrazide **7** and H3K9me2 peptide hydrazide **12** were obtained as white solids (31.7 mg, 7.35 µmol, 17% for peptide **7**; 12.8 mg, 2.98 μmol, 6.0% for peptide **12**). 

Then, H3K4me3 and H3K9me2 peptide hydrazides (5 mg each, final conc., 3.0 mM) were dissolved in 6 M Gn·HCl and 0.2 M NaH_2_PO_4_ at pH 3.0. The solution was cooled to −15 °C and 1.0 M NaNO_2_ aq. was added (10 equiv. against peptide). After 15 min, MESNa (100 equiv. against peptide) was added to the reaction mixture. The pH was adjusted to 6.5–7.0 with 6 N NaOH aq. The peptide solution was purified by HPLC and identified by MALDI-TOF mass spectrometry. After lyophilization, H3K4me3 peptide thioester **8** and H3K9me2 peptide thioester **13** were obtained as white solids (1.10 mg, 0.25 µmol, 41% for peptide **8**; 0.95 mg, 0.22 µmol, 36% for peptide **13**).

#### 3.6.2. Preparation of Histone H3 C-Terminal Peptide Segment by *E. coli* Expression

The DNA fragment encoding Arabidopsis thaliana H3.1 mutant (Ala29Cys) with the recognition sequence of enterokinase (Asp-Asp-Asp-Asp-Lys) was inserted into pET-15b (Novagen, Houston, TX, USA). Notably, the DNA fragment for the recognition sequence of enterokinase was integrated between residues Ser28 and Cys29 of H3.1. The recombinant histone H3.1 mutant was produced as N-terminally hexa-histidine (His_6_)-tagged proteins in Escherichia coli BL21 (DE3) harboring the minor tRNA expression vector (Codon (+) RIL, Stratagene, San Diego, CA, USA). Bacterial cultures were grown on LB plates containing ampicillin (100 µg/mL) at 37 °C for 16 h, then inoculated into TB medium containing ampicillin (100 µg/mL) and cultured at 37 °C. Expression of H3.1 mutant was induced by the addition of 0.5 mM isopropyl-β-D-thiogalactopyranoside when the cell density reached A600 = 0.4–0.6, followed by culture at 37 °C for 12 h. Purification of the H3.1 mutant was performed using a modified version of the previously described method [45,46]. Briefly, His_6_-tagged H3.1 mutant protein was isolated and purified under denaturing conditions using nickel–nitrilotriacetic acid agarose (Ni–NTA) resin (QIAGEN, Hilden, Germany). The enterokinase light chain (New England BioLabs, Ipswich, MA, USA) was added to the purified H3.1 mutant protein (0.57 ng/mg of H3.1 mutant) to remove the His_6_-tagged N-terminal region (aa 1–28), and the mixture was incubated at 20 °C for 16 h. The reaction product was then mixed with 1.5 times the volume of cation exchange buffer (20 mM NaOAc pH5.2, 6 M urea, 10 mM 2-mercaptoethanol, 1 mM EDTA) and subjected to a HiPrep SP HP 16/10 cation exchange column (Cytiva, Muskegon, MI, USA). The truncated H3.1 (aa 29–135) was then eluted by a linear gradient of 0–900 mM NaCl with 20 column volumes of cation exchange buffer. The purified truncated H3.1 was dialyzed four times against water, frozen in liquid nitrogen, and then lyophilized. Then, the truncated H3.1 was purified by preparative HPLC. To convert the N-terminal thiazolidine impurity to the desired N-terminal Cys peptide, to peptide **9** (final conc., 1 mM) dissolved in denaturing buffer (0.2 M NaH_2_PO_4_ [pH 5], 6 M Gn·HCl) was added MeONH_2_·HCl (final conc., 200 mM) and TCEP (final conc., 20 mM). The reaction solution was incubated at 37 °C (pH 5). After 15 h, the reaction solution was purified by HPLC and identified by MALDI-TOF mass spectrometry. After lyophilization, peptide **9** was obtained as a white solid (19.3 mg).

#### 3.6.3. One-Pot NCL/Desulfurization for Full-Length H3K4me3 and H3K9me2

To H3K4me3 peptide thioester **8** (0.37 mg, 84 nmol), peptide **9** (1.04 mg, 70 nmol), and 2.0 µL TCEP solution (500 mM) in degassed NCL buffer (0.2 M NaH_2_PO_4_ [pH 7], 6 M Gn·HCl; 50 μL) were added thiocholine (4.0 µmol). The reaction solution was incubated at 37 °C (pH 7). After 4 h, to the reaction solution was added TCEP (17.2 mg, 60 µmol) and VA-044 (1.29 mg, 4 µmol), and the mixture was diluted with NCL buffer for desulfurization (sample volume, 200 µL; pH 7). The final content of the mixture was peptide (0.35 mM), TCEP (300 mM), thiocholine (20 mM), and VA-044 (20 mM). For analysis of each reaction, 0.5 μL of reaction solution was taken and quenched with NCL buffer (pH 3.0, 44 μL) and 500 mM TCEP aq. (5.0 μL), followed by injection on an analytical HPLC equipped with a 4.6 ID × 250 mm Jupiter 5C4 300Å column (Phenomenex). HPLC peaks were monitored at 220 nm in a linear gradient with water/acetonitrile containing 0.1% TFA (gradient: 5–75% for 30 min). After reaction completion, the reaction solution was purified by preparative HPLC and identified by MALDI-TOF MS. After lyophilization, peptide **11** was obtained as a white solid (0.57 mg, 30.0 µmol, 43%).

To H3K9me2 peptide thioester **13** (0.41 mg, 96 nmol), peptide **9** (1.19 mg, 80 nmol), and 1.6 µL TCEP solution (500 mM) in degassed NCL buffer (0.2 M NaH_2_PO_4_ [pH 7], 6 M Gn·HCl; 50 μL) were added thiocholine (3.2 µmol). The reaction solution was incubated at 37 °C (pH 7). After 4 h, to the reaction solution was added TCEP (8.6 mg, 30 µmol) and VA-044 (0.64 mg, 2.0 µmol), and the mixture was diluted with NCL buffer for desulfurization (sample volume, 100 µL; pH 7). After 46 h, additional TCEP (17.2 mg, 60 µmol) and VA-044 (1.28 mg, 4.0 µmol) were added, and the reaction solution was diluted with NCL buffer (sample volume, 200 µL; pH 7) to complete the reaction. The final content of the mixture was peptide (0.4 mM), TCEP (300 mM), thiocholine (16 mM), and VA-044 (20 mM). For analysis of each reaction, 0.5 μL of reaction solution was taken and quenched with NCL buffer (pH 3.0, 44 μL) and 500 mM TCEP aq. (5.0 μL), followed by injection on an analytical HPLC equipped with a 4.6 ID × 250 mm Jupiter 5C4 300Å column (Phenomenex). HPLC peaks were monitored at 220 nm in a linear gradient with water/acetonitrile containing 0.1% TFA (gradient: 5–75% for 30 min). After reaction completion, the reaction solution was purified by preparative HPLC and identified by MALDI-TOF MS. After lyophilization, peptide **15** was obtained as a white solid (0.40 mg, 20.8 µmol, 26%).

## 4. Conclusions

In this study, we proposed and demonstrated thiocholine as a new thiol catalyst/additive in NCL and desulfurization, which are two key reactions in chemical protein synthesis. Our results revealed that thiocholine peptide thioester exhibited practical NCL reactivity and moderate hydrolytic stability among several alkyl thioesters, indicating that these properties reflected the pKa of thiocholine (~7.8). To our surprise, this thiol compound showed better activity in desulfurization than MTG, which has a similar pKa value as thiocholine. Finally, we demonstrated semisynthesis of Arabidopsis histone H3K4me3 and H3K9me2 through thiocholine-mediated one-pot NCL/desulfurization. Thiocholine is expected to be a useful option in the field of chemical protein synthesis because of its high hydrophilicity and odorless nature.

## Data Availability

Not applicable.

## Supplementary Materials

The following supporting information can be downloaded at: https://www.mdpi.com/article/10.3390/molecules28093655/s1, Scheme S1: Synthetic scheme of thiocholine chloride; Figure S1: Synthesis of model peptide thioesters; Figure S2: Synthesis of model peptides **2**, **3**, and **4**; Figure S3: Time-course HPLC analyses of hydrolysis reactions with each peptide thioester; Figure S4: Time-course HPLC analyses of NCL reactions between each model peptide thioester and peptide **3**; Figure S5: Synthesis of peptide **5**; Figure S6: MALDI-TOF mass spectrum of peptide **6**; Figure S7: Time-course HPLC analyses of model peptide desulfurization with different thiol additives; Figure S8: Time-course HPLC analyses of model peptide desulfurization with different concentrations of thiocholine; Figure S9: Synthesis of H3K4me3 N-terminal peptide **8**; Figure S10: Preparation of H3 C-terminal peptide **9**; Figure S11: MALDI-TOF mass spectrum of peptide **10**; Figure S12: Synthesis of H3K9me2 N-terminal peptide **13**; Figure S13: Semisynthesis of Arabidopsis H3K9me2 via thiocholine-mediated one-pot NCL/desulfurization.

## References

[1] Dawson P.E., Muir T.W., Clark-Lewis I., Kent S.B.H. (1994). Synthesis of Proteins by Native Chemical Ligation. Science.

[2] Agouridas V., Mahdi O.E., Diemer V., Cargoet M., Monbaliu J.C.M., Melnyk O. (2019). Native Chemical Ligation and Extended Methods: Mechanisms, Catalysis, Scope, and Limitations. Chem. Rev..

[3] Kulkarni S.S., Sayers J., Premdjee B., Payne R.J. (2018). Rapid and efficient protein synthesis through expansion of the native chemical ligation concept. Nat. Rev. Chem..

[4] Conibear A.C., Watson E.E., Payne R.J., Becker C.F.W. (2018). Native chemical ligation in protein synthesis and semi-synthesis. Chem. Soc. Rev..

[5] Diemer V., Firstova O., Agouridas V., Melnyk O. (2022). Pedal to the Metal: The Homogeneous Catalysis of the Native Chemical Ligation Reaction. Chem. Eur. J..

[6] Hupe D.J., Jencks W.P. (1977). Nonlinear structure-reactivity correlations. Acyl transfer between sulfur and oxygen nucleophiles. J. Am. Chem. Soc..

[7] Johnson E.C.B., Kent S.B.H. (2006). Insights into the Mechanism and Catalysis of the Native Chemical Ligation Reaction. J. Am. Chem. Soc..

[8] Wan Q., Danishefsky S.J. (2007). Free-Radical-Based, Specific Desulfurization of Cysteine: A Powerful Advance in the Synthesis of Polypeptides and Glycopolypeptides. Angew. Chem. Int. Ed..

[9] Jin K., Li X. (2018). Advances in Native Chemical Ligation–Desulfurization: A Powerful Strategy for Peptide and Protein Synthesis. Chem. Eur. J..

[10] Evans T.C., Benner J., Xu M.Q. (1998). Semisynthesis of cytotoxic proteins using a modified protein splicing element. Protein Sci..

[11] Siman P., Blatt O., Moyal T., Danieli T., Lebendiker M., Lashuel H.A., Friedler A., Brik A. (2011). Chemical Synthesis and Expression of the HIV-1 Rev Protein. ChemBioChem.

[12] Shimko J.C., North J.A., Bruns A.N., Poirier M.G., Ottesen J.J. (2011). Preparation of Fully Synthetic Histone H3 Reveals That Acetyl-Lysine 56 Facilitates Protein Binding Within Nucleosomes. J. Mol. Biol..

[13] Hayashi G., Sueoka T., Okamoto A. (2016). In vitro and in cell analysis of chemically synthesized histone H2A with multiple modifications. Chem. Commun..

[14] Huang Y.C., Chen C.C., Gao S., Wang Y.H., Xiao H., Wang F., Tian C.L., Li Y.M. (2016). Synthesis of L- and D-Ubiquitin by One-Pot Ligation and Metal-Free Desulfurization. Chem. Eur. J..

[15] Thompson R.E., Liu X., Alonso-García N., Pereira P.J.B., Jolliffe K.A., Payne R.J. (2014). Trifluoroethanethiol: An Additive for Efficient One-Pot Peptide Ligation-Desulfurization Chemistry. J. Am. Chem. Soc..

[16] Ohkawachi K., Kobayashi D., Morimoto K., Shigenaga A., Denda M., Yamatsugu K., Kanai M., Otaka A. (2020). Sulfanylmethyldimethylaminopyridine as a Useful Thiol Additive for Ligation Chemistry in Peptide/Protein Synthesis. Org. Lett..

[17] Bagiyan G.A., Grachev S.A., Koroleva I.K., Soroka N.V. (1976). The kinetics and mechanism of the reaction of the oxidation of aminothiols by hydrogen peroxide in aqueous solutions. Bull. Acad. Sci. USSR Div. Chem. Sci..

[18] Chalker J.M., Lercher L., Rose N.R., Schofield C.J., Davis B.G. (2012). Conversion of Cysteine into Dehydroalanine Enables Access to Synthetic Histones Bearing Diverse Post-Translational Modifications. Angew. Chem. Int. Ed..

[19] Fang G.M., Li Y.M., Shen F., Huang Y.C., Li J.B., Lin Y., Cui H.K., Liu L. (2011). Protein Chemical Synthesis by Ligation of Peptide Hydrazides. Angew. Chem. Int. Ed..

[20] Zheng J.S., Tang S., Qi Y.K., Wang Z.P., Liu L. (2013). Chemical Synthesis of proteins using peptide hydrazides as thioester surrogates. Nat. Protcol..

[21] Hackeng T.M., Griffin J.H., Dawson P.E. (1999). Protein synthesis by native chemical ligation: Expanded scope by using straightforward methodology. Proc. Natl. Acad. Sci. USA.

[22] Gao X.F., Du J.J., Liu Z., Guo J. (2016). Visible-Light-Induced Specific Desulfurization of Cysteinyl Peptide and Glycopeptide in Aqueous Solution. Org. Lett..

[23] Lee M., Neukirchen S., Cabrele C., Reiser O. (2017). Visible-light photoredox-catalyzed desulfurization of thiol- and disulfide-containing amino acids and small peptides. J. Pept. Sci..

[24] Chisholm T.S., Clayton D., Dowman L.J., Sayers J., Payne R.J. (2018). Native Chemical Ligation–Photodesulfurization in Flow. J. Am. Chem. Soc..

[25] Xu L., Xu Y., Qu Q., Guan C.J., Chu G.C., Shi J., Li Y.M. (2016). Efficient chemical synthesis for the analogue of ubiquitin-based probe Ub–AMC with native bioactivity. RSC Adv..

[26] Erickson P.W., Fulcher J.M., Spaltenstein P., Kay M.S. (2021). Traceless Click-Assisted Native Chemical Ligation Enabled by Protecting Dibenzocyclooctyne from Acid-Mediated Rearrangement with Copper(I). Bioconjug. Chem..

[27] Pan B., Park J.H., Ramlall T., Eliezer D., Rhoades E., Petersson E.J. (2021). Chemoenzymatic Semi-synthesis Enables Efficient Production of Isotopically Labeled α-Synuclein with Site-Specific Tyrosine Phosphorylation. ChemBioChem.

[28] Tian Y., Wang L., Shi J., Yu H.Z. (2015). Desulfurization Mechanism of Cysteine in Synthesis of Polypeptides. Chin. J. Chem. Phys..

[29] Qiu W., Shi S., Li R., Lin X., Rao L., Sun Z. (2021). A Mild, General, Metal-Free Method for Desulfurization of Thiols and Disulfides Induced by Visible-Light. Chin. J. Chem..

[30] Venneti N.M., Samala G., Morsy R.M.I., Mendoza L.G., Isidro-Llobet A., Tom J.K., Mukherjee S., Kopach M.E., Stockdill J.L. (2023). Phosphine-Dependent Photoinitiation of Alkyl Thiols under Near-UV Light Facilitates User-Friendly Peptide Desulfurization. J. Am. Chem. Soc..

[31] Sun Z., Ma W., Cao Y., Wei T., Mo X., Chow H.T., Tan Y., Cheung C.H.P., Liu J., Lee H.K. (2022). Superfast desulfurization for protein chemical synthesis and modification. Chem.

[32] Roudier F., Ahmed I., Bérard C., Sarazin A., Mary-Huard T., Cortijo S., Bouyer D., Caillieux E., Duvernois-Berthet E., Al-Shikhley L. (2011). Integrative epigenomic mapping defines four main chromatin states in Arabidopsis. EMBO J..

[33] Oh S., Park S., van Nocker S. (2008). Genic and Global Functions for Paf1C in Chromatin Modification and Gene Expression in Arabidopsis. PLoS Genet..

[34] Houden A., Demidov D., Gernand D., Meister A., Leach C.R., Schubert I. (2003). Methylation of histone H3 in euchromatin of plant chromosomes depends on basic nuclear DNA content. Plant J..

[35] Jiang D., Kong N.C., Gu X., Li Z., He Y. (2011). *Arabidopsis* COMPASS-Like Complexes Mediate Histone H3 Lysine-4 Trimethylation to Control Floral Transition and Plant Development. PLoS Genet..

[36] Zhang X., Bernatavichute Y.V., Cokus S., Pellegrini M., Jacobsen S.E. (2009). Genome-wide analysis of mono-, di- and trimethylation of histone H3 lysine 4 in Arabidopsis thaliana. Genome Biol..

[37] Bang D., Kent S.B.H. (2004). A One-Pot Total Synthesis of Crambin. Angew. Chem. Int. Ed..

[38] Xu L., Jiang H. (2020). Writing and Reading Histone H3 lysine 9 Methylation in Arabidopsis. Front. Plant Sci..

[39] Jackson J.P., Johnson L., Jasencakova Z., Zhang X., PerezBurgos L., Singh P.B., Cheng X., Schubert I., Jenuwein T., Jacobsen S.E. (2004). Dimethylation of histone H3 lysine 9 is a critical mark for DNA methylation and gene silencing in Arabidopsis thaliana. Chromosoma.

[40] Soppe W.J.J., Jasencakova Z., Houben A., Kakutani T., Meister A., Huang M.S., Jacobsen S.E., Schubert I., Fransz P.F. (2002). DNA methylation controls histone H3 lysine 9 methylation and heterochromatin assembly in Arabidopsis. EMBO J..

[41] Jasencakova Z., Soppe W.J.J., Meister A., Gernand D., Turner B.M., Schubert I. (2003). Histone modifications in Arabidopsis—High methylation of H3 lysine 9 is dispensable for constitutive heterochromatin. Plant J..

[42] Naumann K., Fischer A., Hofmann I., Krauss V., Phalke S., Irmler K., Hause G., Aurich A.C., Dorn R., Jenuwein T. (2005). Pivotal role of AtSUVH2 in heterochromatic histone methylation and gene silencing in Arabidopsis. EMBO J..

[43] Zhang C., Du X., Tang K., Yang Z., Pan L., Zhu P., Luo J., Jiang Y., Zhang H., Wan H. (2018). Arabidopsis AGDP1 links H3K9me2 to DNA methylation in heterochromatin. Nat. Commun..

[44] Walker B.J., Nair G.P., Marshall L.F., Bulović V., Bawendi M.G. (2009). Narrow-Band Absorption-Enhanced Quantum Dot/J-Aggregate Conjugates. J. Am. Chem. Soc..

[45] Tachiwana H., Kagawa W., Osakabe A., Kurumizaka H. (2010). Structural basis of instability of the nucleosome containing a testis-specific histone variant, human H3T. Proc. Natl. Acad. Sci. USA.

[46] Kujirai T., Arimura Y., Fujita R., Horikoshi N., Machida S., Kurumizaka H., Orsi G., Almouzni G. (2018). Methods for Preparing Nucleosomes Containing Histone Variants.

