# Supplementary material for: Thiocholine-Mediated One-Pot Peptide Ligation and Desulfurization
Source: Molecules. 2023 Apr 22;28(9):3655. doi: 10.3390/molecules28093655 (PMC10179797; doi:10.3390/molecules28093655)
Supplement: Supplementary file 1 [file molecules-28-03655-s001.zip › molecules-2347279-supplementary.pdf]

Supplementary material

Thiocholine-Mediated One-Pot Peptide Ligation and Desulfurization

Contents

Scheme S1. Synthetic scheme of thiocholine chloride..........................2

Figure S1. Synthesis of model peptide thioesters..........................3

Figure S2. Synthesis of model peptides **2**, **3**, and **4**..........................4

Figure S3. Time course HPLC analyses of hydrolysis reaction with each peptide thioester.........5

Figure S4. Time course HPLC analyses of NCL reaction between each model peptide thioester and peptide **3**..........................6

Figure S5. Synthesis of peptides **5**..........................7

Figure S6. MALDI-TOF mass spectrum of peptide **6**..........................8

Figure S7. Time course HPLC analyses of model peptide desulfurization with different thiol additives..........................9

Figure S8. Time course HPLC analyses of model peptide desulfurization with different concentration of thiocholine..........................10

Figure S9. Synthesis of H3K4me3 N-terminal peptide **8**..........................11

Figure S10. Preparation of H3 C-terminal peptide **9**..........................12

Figure S11. MALDI-TOF mass spectrum of peptide **10**..........................13

Figure S12. Synthesis of H3K9me2 N-terminal peptide **13**..........................14

Figure S13. Semisynthesis of Arabidopsis H3K9me2 via thiocholine-mediated one-pot NCL/desulfurization..........................15

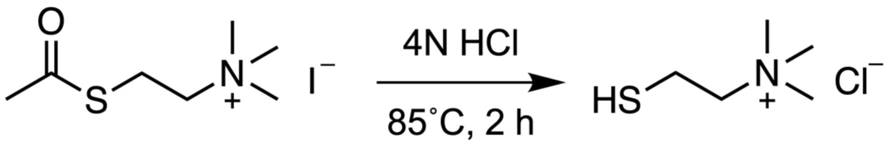

Scheme S1. Synthetic scheme of thiocholine chloride.

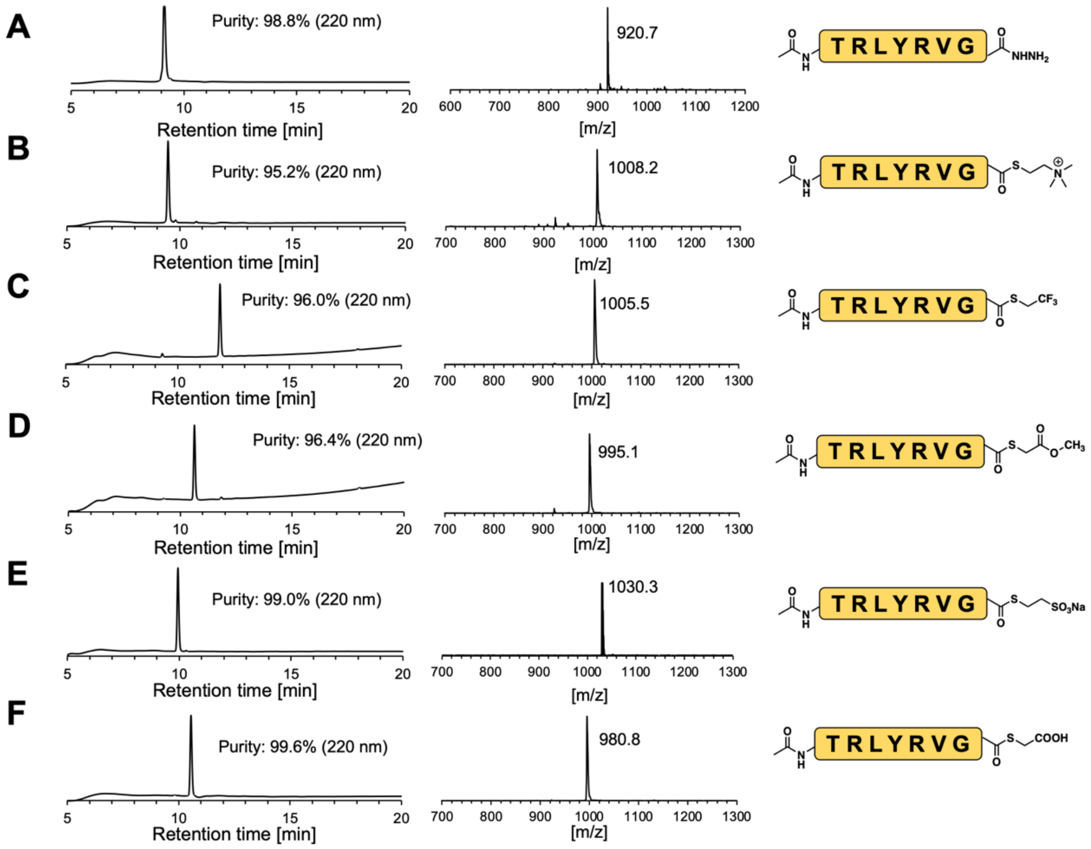

Figure S1. Synthesis of model peptide thioesters. HPLC charts and MALDI-TOF mass spectra of purified model peptide thioesters are shown. HPLC peaks were monitored at 220 nm in the linear gradient with water/acetonitrile containing 0.1% TFA. Gradient: 10–70% for 20 min with 5C18-AR-II column. (A) **Peptide hydrazide**; Calculated mass $[M+H]^+$: 920.5; Mass Found $[M+H]^+$: 920.7. (B) **Peptide thiocholine thioester 1**; Calculated mass $[M]^+$: 1007.6; Mass Found $[M]^+$: 1008.2. (C) **Peptide TFET thioester**; Calculated mass $[M+H]^+$: 1005.1; Mass Found $[M+H]^+$: 1005.5. (D) **Peptide MTG thioester**; Calculated mass $[M+H]^+$: 994.5; Mass Found $[M+H]^+$: 995.1. (E) **Peptide MESNa thioester**; Calculated mass $[M+H]^+$: 1030.5; Mass Found $[M+H]^+$: 1030.3. (F) **Peptide TGA thioester**; Calculated mass $[M+H]^+$: 981.1; Mass Found $[M+H]^+$: 980.8.

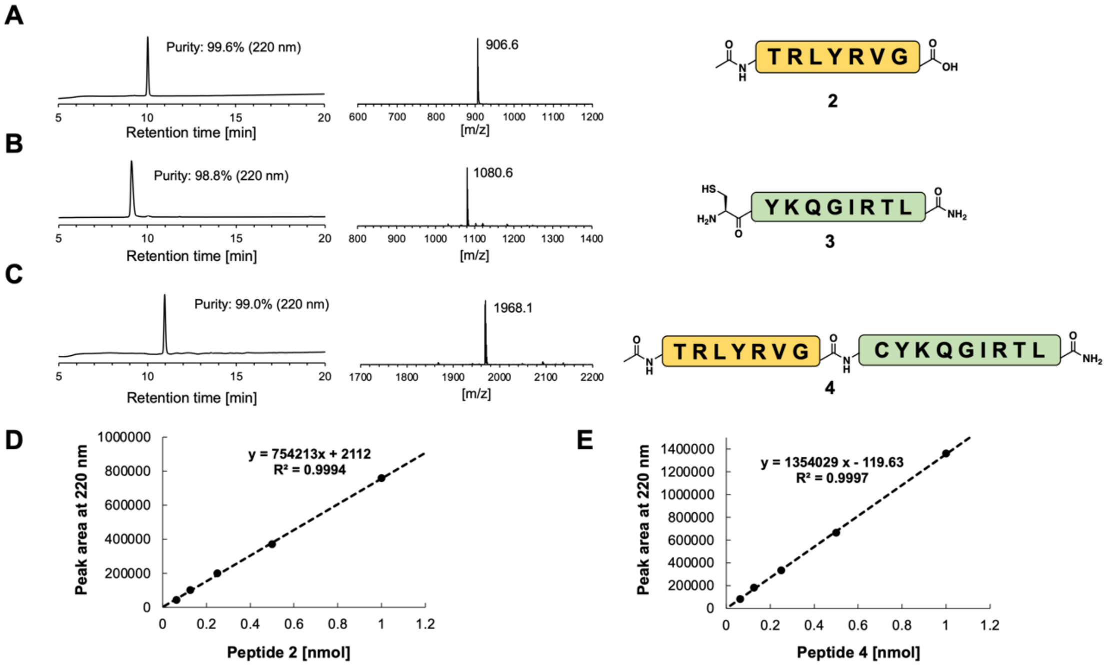

Figure S2. Synthesis of model peptides **2**, **3**, and **4**. HPLC charts and MALDI-TOF mass spectra of purified peptides **2**, **3**, and **4**. HPLC peaks were monitored at 220 nm in the linear gradient with water/acetonitrile containing 0.1% TFA. Gradient: 10–70% for 20 min with 5C18-AR-II column. (A) **2**; Calculated mass $[M+H]^+$: 906.5; Mass Found $[M+H]^+$: 906.6. (B) **3**; Calculated mass $[M+H]^+$: 1080.6; Mass Found $[M+H]^+$: 1080.6. (C) **4**; Calculated mass $[M+H]^+$: 1968.1; Mass Found $[M+H]^+$: 1968.1. (D) Calibration curve of peptide **2**. (E) Calibration curve of peptide **4**.

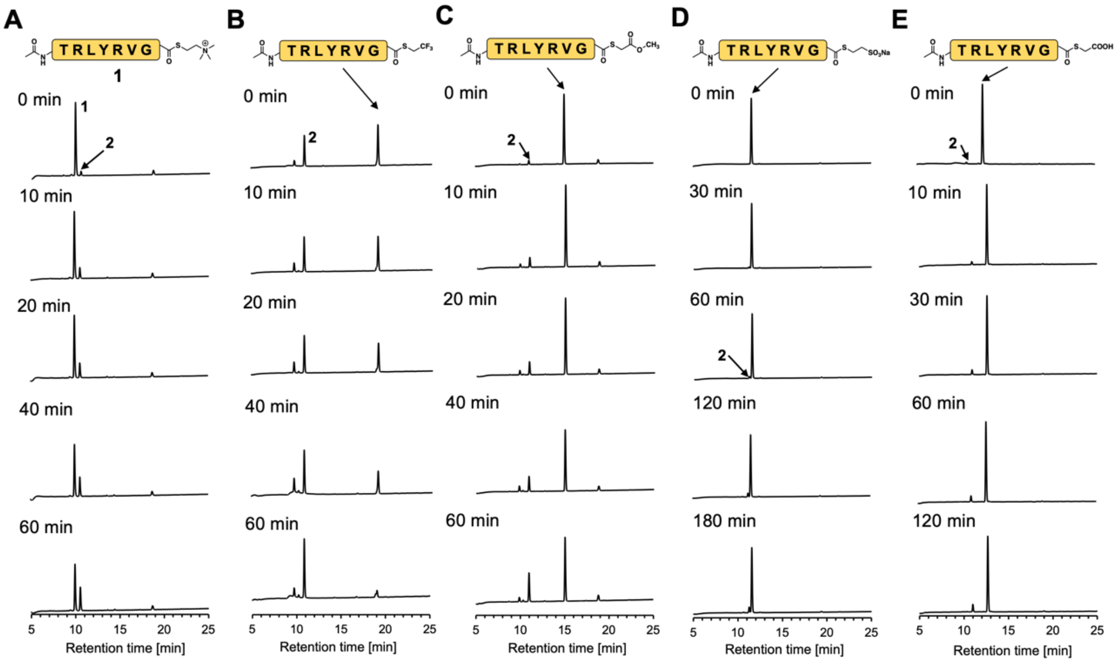

Figure S3. Time course HPLC analyses of hydrolysis reaction with each peptide thioester. Each peptide (1 mM) of (A) thiocholine thioester **1**, (B) TFET thioester, (C) MTG thioester, (D) MESNa thioester, or (E) TGA thioester was incubated in denaturing buffer (0.2 M NaH_2PO_4, 6 M Gn·HCl, 40 mM TCEP) at pH 7.0, 25°C. HPLC peaks were monitored at 220 nm in the linear gradient with water/acetonitrile containing 0.1% TFA. Gradient: 15–50% for 30 min with 5C18-AR-II column.

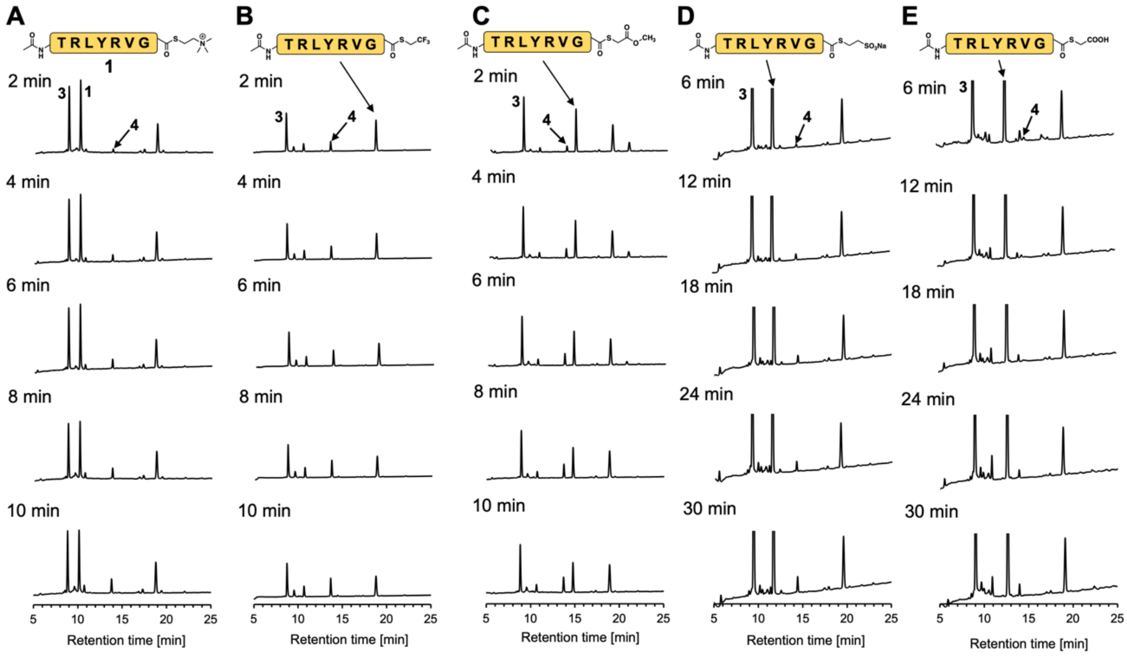

Figure S4. Time course HPLC analyses of NCL reaction between each model peptide thioester and peptide **3**. Each model peptide (0.1 mM) of (A) thiocholine thioester **1**, (B) TFET thioester, (C) MTG thioester, (D) MESNa thioester, or (E) TGA thioester, and peptide **3** were incubated in denaturing buffer (0.2 M NaH_2PO_4, 6 M Gn·HCl, 40 mM TCEP) at pH 7.0, 25℃. HPLC peaks were monitored at 220 nm in the linear gradient with water/acetonitrile containing 0.1% TFA. Gradient: 15–50% for 30 min with 5C18-AR-II column.

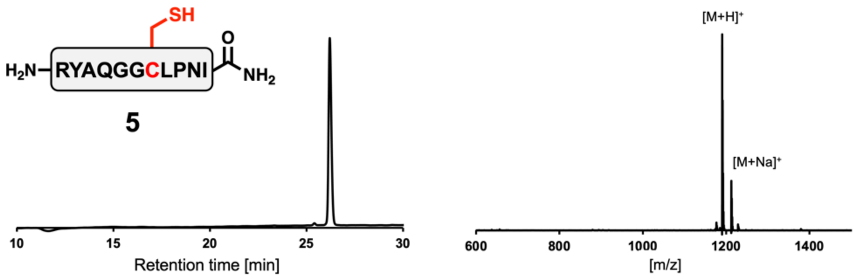

Figure S5. Synthesis of peptides **5**. A HPLC chart and MALDI-TOF mass spectrum of purified peptide **5**. HPLC peaks were monitored at 220 nm in the linear gradient with water/acetonitrile containing 0.1% TFA. Gradient: 5–30% for 30 min with 5C18-AR-II column. Calculated mass $[M+H]^+$: 1190.6; Mass Found $[M+H]^+$: 1190.6.

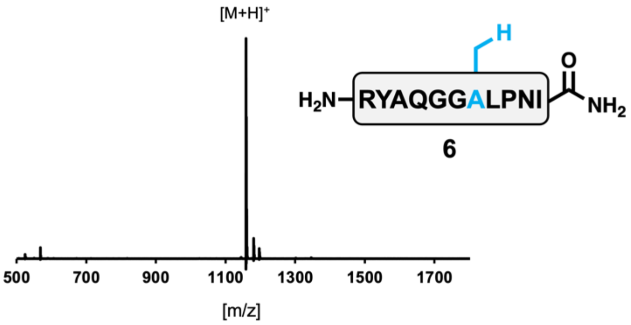

Figure S6. MALDI-TOF mass spectrum of peptide **6**. Calculated mass $[M+H]^+$: 1158.6; Mass Found $[M+H]^+$: 1158.7.

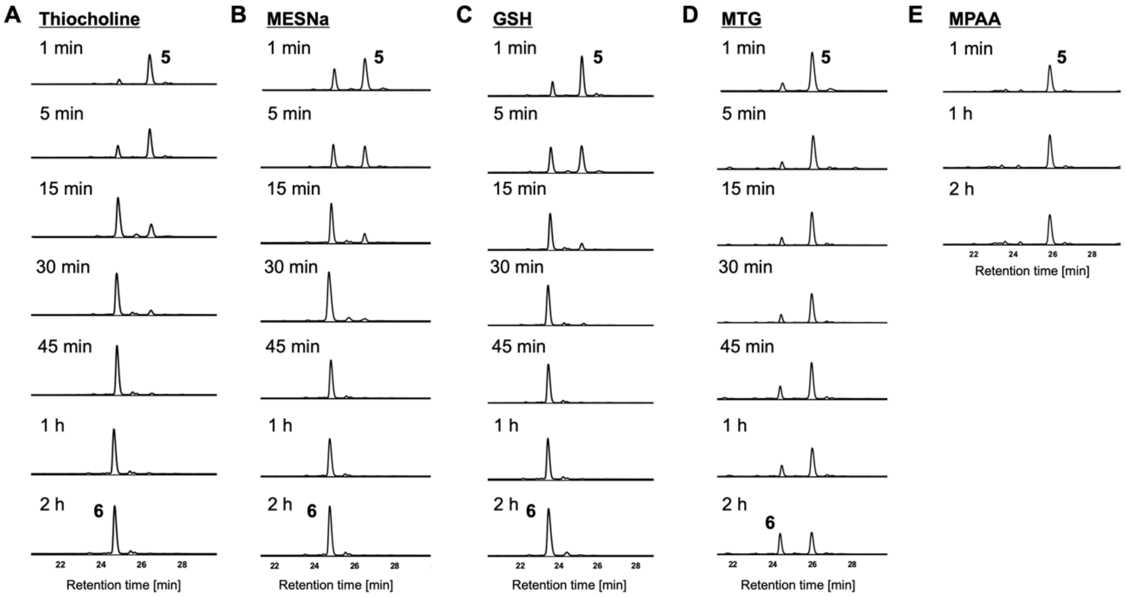

Figure S7. Time course HPLC analyses of model peptide desulfurization with different thiol additives. Peptide **5** (0.8 mM) and 80 mM thiol additives, (A) thiocholine, (B) MESNa, (C) GSH, (D) MTG, or (E) MPAA (80 mM), TCEP (300 mM), and VA-044 (20 mM) were reacted in denaturing buffer (0.2 M NaH_2PO_4, 6 M Gn·HCl) at pH 7.0, 37°C. The reaction was quenched with 100 mM ascorbic acid before HPLC analyses. HPLC peaks were monitored at 220 nm in the linear gradient with water/acetonitrile containing 0.1% TFA. Gradient: 5–30% for 30 min with 5C18-AR-II column.

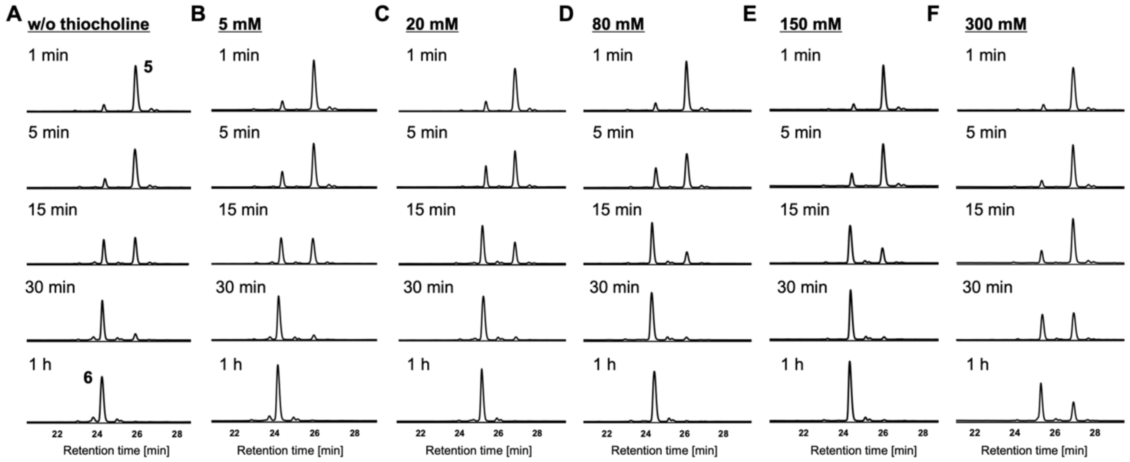

Figure S8. Time course HPLC analyses of model peptide desulfurization with different concentration of thiocholine. Peptide **5** (0.8 mM) and thiocholine (A) 0 mM, (B) 5 mM, (C) 20 mM, (D) 80 mM, (E) 150 mM, or (F) 300 mM, TCEP (300 mM), and VA-044 (20 mM) were reacted in denaturing buffer (0.2 M NaH_2PO_4, 6 M Gn·HCl) at pH 7.0, 37℃. The reaction was quenched with 100 mM ascorbic acid before HPLC analyses. HPLC peaks were monitored at 220 nm in the linear gradient with water/acetonitrile containing 0.1% TFA. Gradient: 5–30% for 30 min with 5C18 AR-II.

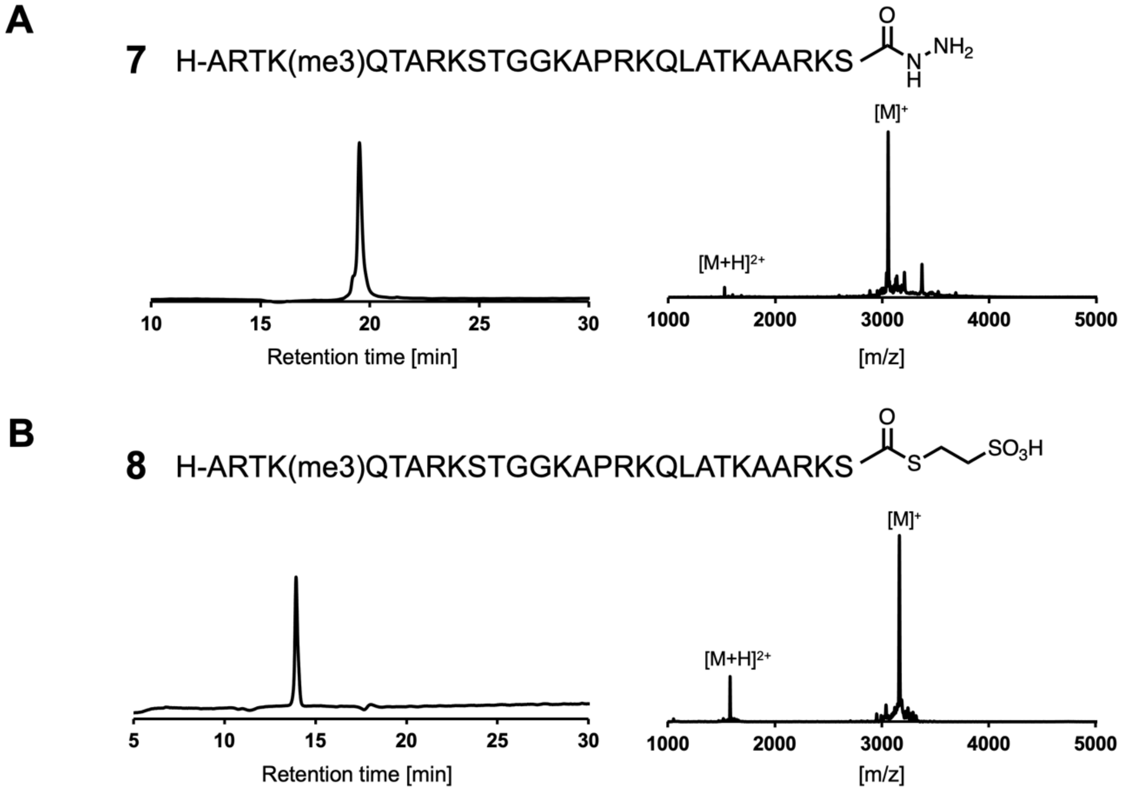

Figure S9. Synthesis of H3K4me3 N-terminal peptide **8**. HPLC charts and MALDI-TOF mass spectra of purified peptide **7** (A) and peptide **8** (B). HPLC peaks were monitored at 220 nm in the linear gradient with water/acetonitrile containing 0.1% TFA. (A) Gradient: 2–25% for 30 min with 5C18-AR-II column. (B) Gradient: 5–30% for 30 min with 5C18-AR-II column. Calculated mass of **7** $[M]^+$: 3054.6; Mass Found $[M]^+$: 3054.8. Calculated mass of **8** $[M]^+$: 3164.7; Mass Found $[M]^+$: 3164.8.

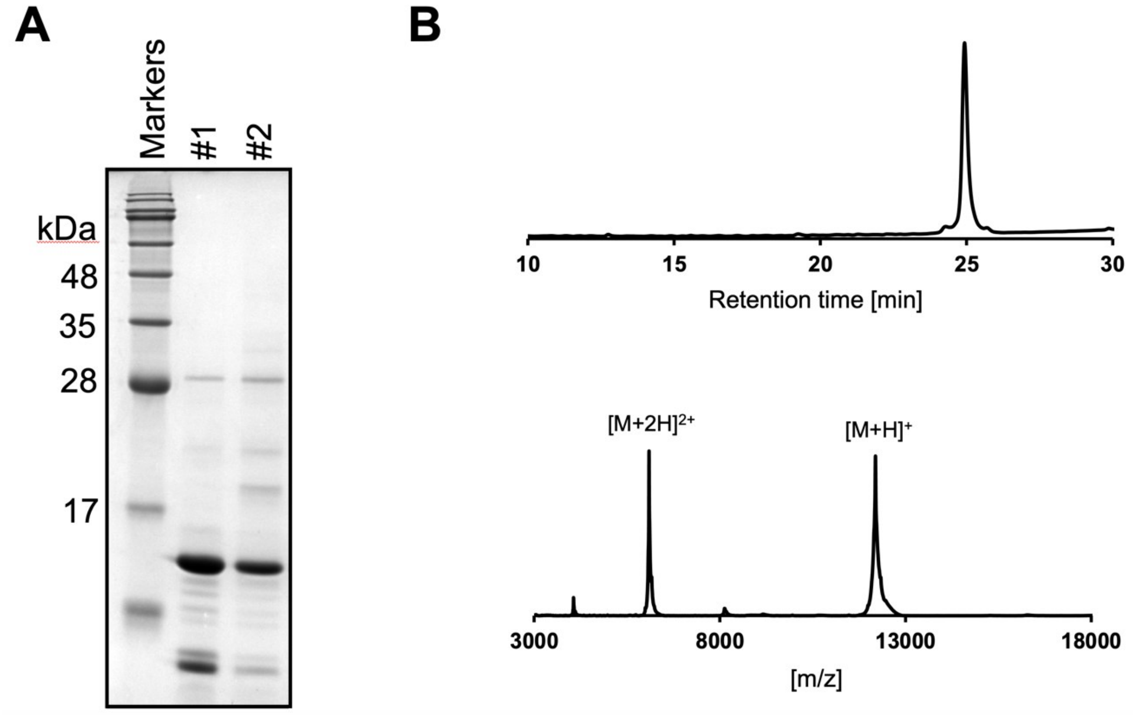

Figure S10. Preparation of H3 C-terminal peptide **9**. (A) SDS-PAGE of peptide **9** before HPLC purification. (B) HPLC chart and MALDI-TOF mass spectrum of peptide **9** after HPLC purification. HPLC peaks were monitored at 220 nm in the linear gradient with water/acetonitrile containing 0.1% TFA. Gradient: 20–70% for 30 min with Jupiter 5C4 column. Calculated mass of **9** $[M+H]^+$: 12190.1; Mass Found $[M+H]^+$: 12190.2.

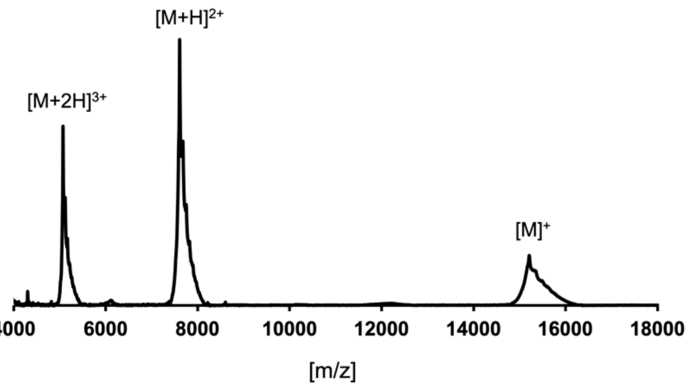

Figure S11. MALDI-TOF mass spectrum of peptide **10**. Calculated mass of **10** [M]$^+$: 15211.7; Mass Found [M]$^+$: 15210.7.

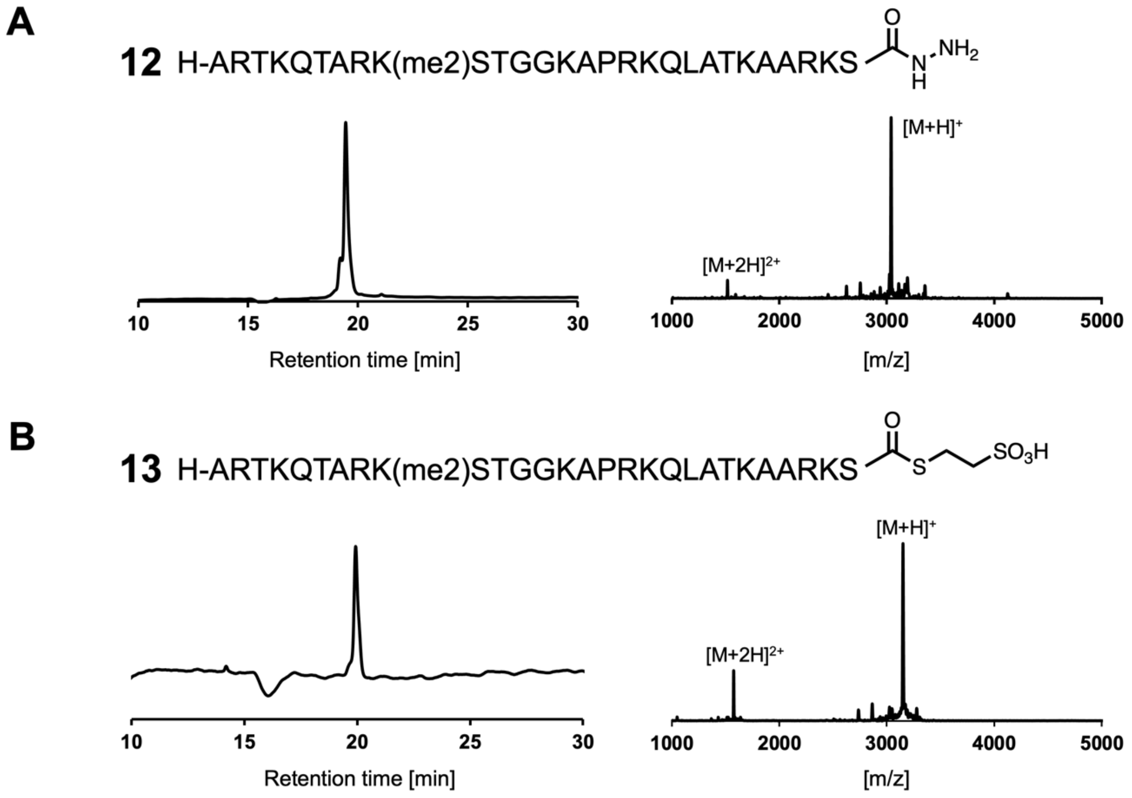

Figure S12. Synthesis of H3K9me2 N-terminal peptide **13**. HPLC charts and MALDI-TOF mass spectra of purified peptide **12** (A) and peptide **13** (B). HPLC peaks were monitored at 220 nm in the linear gradient with water/acetonitrile containing 0.1% TFA. (A) Gradient: 2–25% for 30 min with 5C18-AR-II column. (B) Gradient: 5–30% for 30 min with 5C18-AR-II column. Calculated mass of **12** $[M+H]^+$: 3040.6; Mass Found $[M]^+$: 3040.2. Calculated mass of **13** $[M+H]^+$: 3150.7; Mass Found $[M]^+$: 3150.9.

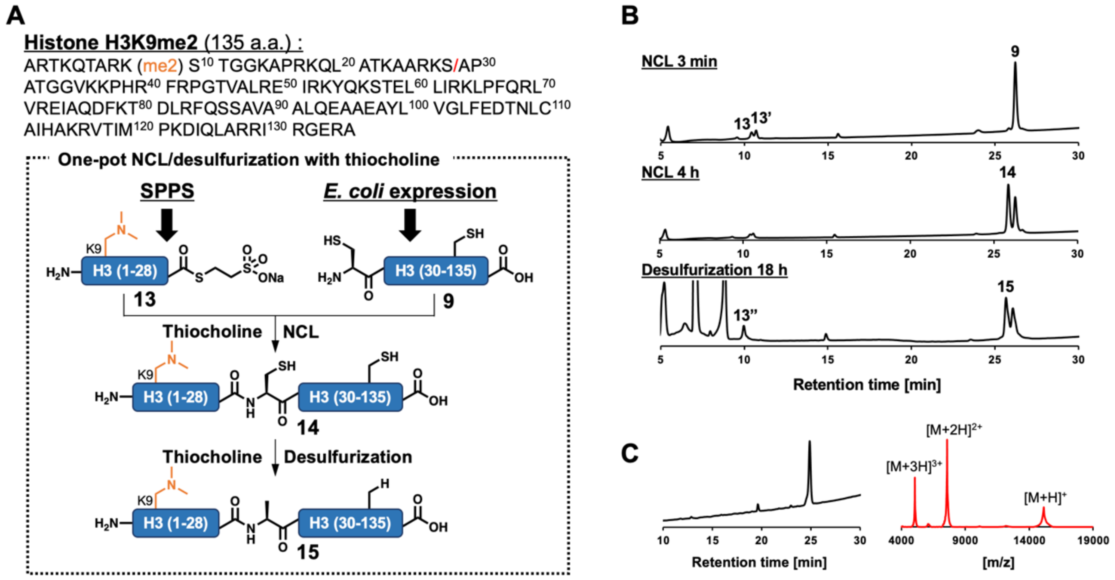

Figure S13. Semisynthesis of Arabidopsis H3K9me2 via thiocholine-mediated one-pot NCL/desulfurization. (A) Amino-acid sequence and synthetic scheme of H3K9me2. A red slash represents the ligation junction. (B) Reaction tracking of the one-pot NCL/desulfurization by analytical HPLC (gradient: 5–75% for 30 min) at 220 nm. Compounds **13'** and **13''** are thiocholine thioester and hydrolysis of peptides **13**, respectively. HPLC profiles at NCL at 3 min, NCL at 4 h and desulfurization at 18 h were shown. (C) HPLC profile (left, gradient 20–70% for 30 min) and MALDI-TOF mass spectrum (right) of purified peptide **15**. Calculated mass of **15** $[M+H]^+$: 15133.5; mass found $[M+H]^+$: 15132.8.
